# Simultaneous Determination of 6-Shogaol and 6-Gingerol in Various Ginger (*Zingiber officinale* Roscoe) Extracts and Commercial Formulations Using a Green RP-HPTLC-Densitometry Method

**DOI:** 10.3390/foods9081136

**Published:** 2020-08-18

**Authors:** Ahmed I. Foudah, Faiyaz Shakeel, Hasan S. Yusufoglu, Samir A. Ross, Prawez Alam

**Affiliations:** 1 Department of Pharmacognosy, College of Pharmacy, Prince Sattam Bin Abdulaziz University, Al-Kharj 11942, Saudi Arabia; a.foudah@psau.edu.sa (A.I.F.); h.yusufoglu@psau.edu.sa (H.S.Y.); 2 Department of Pharmaceutics, College of Pharmacy, King Saud University, Riyadh 11451, Saudi Arabia; faiyazs@fastmail.fm; 3 National Center for Natural Products Research, University of Mississippi, Oxford, MS 38677, USA; sroos@olemiss.edu; 4 Department of Biomolecular Sciences, School of Pharmacy, University of Mississippi, Oxford, MS 38677, USA

**Keywords:** 6-gingerol, 6-shogaol, commercial formulation, ginger extract, green reversed phase high-performance thin-layer chromatography (RP-HPTLC), simultaneous analysis

## Abstract

Various analytical methodologies have been reported for the determination of 6-shogaol (6-SHO) and 6-gingerol (6-GIN) in ginger extracts and commercial formulations. However, green analytical methods for the determination of 6-SHO and 6-GIN, either alone or in combination, have not yet been reported in literature. Hence, the present study was aimed to develop a rapid, simple, and cheaper green reversed phase high-performance thin-layer chromatography (RP-HPTLC) densitometry method for the simultaneous determination of 6-SHO and 6-GIN in the traditional and ultrasonication-assisted extracts of ginger rhizome, commercial ginger powder, commercial capsules, and commercial ginger teas. The simultaneous analysis of 6-SHO and 6-GIN was carried out via RP-18 silica gel 60 F254S HPTLC plates. The mixture of green solvents, i.e., ethanol:water (6.5:3.5 *v/v*) was utilized as a mobile phase for the simultaneous analysis of 6-SHO and 6-GIN. The analysis of 6-SHO and 6-GIN was performed at λ_max_ = 200 nm for 6-SHO and 6-GIN. The densitograms of 6-SHO and 6-GIN from traditional and ultrasonication-assisted extracts of ginger rhizome, commercial ginger powder, commercial capsules, and commercial ginger teas were verified by obtaining their single band at R_f_ = 0.36 ± 0.01 for 6-SHO and R_f_ = 0.53 ± 0.01 for 6-GIN, compared to standard 6-SHO and 6-GIN. The green RP-HPTLC method was found to be linear, in the range of 100–700 ng/band with R^2^ = 0.9988 for 6-SHO and 50–600 ng/band with R^2^ = 0.9995 for 6-GIN. In addition, the method was recorded as “accurate, precise, robust and sensitive” for the simultaneous quantification of 6-SHO and 6-GIN in traditional and ultrasonication-assisted extracts of ginger rhizome, commercial ginger powder, commercial capsules, and commercial ginger teas. The amount of 6-SHO in traditional extracts of ginger rhizome, commercial ginger powder, commercial capsules, and commercial ginger teas was obtained as 12.1, 17.9, 10.5, and 9.6 mg/g of extract, respectively. However, the amount of 6-SHO in ultrasonication-assisted extracts of ginger rhizome, commercial ginger powder, commercial capsules, and commercial ginger teas were obtained as 14.6, 19.7, 11.6, and 10.7 mg/g of extract, respectively. The amount of 6-GIN in traditional extracts of ginger rhizome, commercial ginger powder, commercial capsules, and commercial ginger teas were found as 10.2, 15.1, 7.3, and 6.9 mg/g of extract, respectively. However, the amount of 6-GIN in ultrasonication-assisted extracts of ginger rhizome, commercial ginger powder, commercial capsules, and commercial ginger teas were obtained as 12.7, 17.8, 8.8, and 7.9 mg/g of extract, respectively. Overall, the results of this study indicated that the proposed analytical technique could be effectively used for the simultaneous quantification of 6-SHO and 6-GIN in a wide range of plant extracts and commercial formulations.

## 1. Introduction

**Figure 1:**
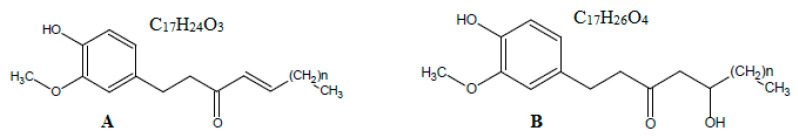
Chemical structure of (**A**) 6-shogaol (6-SHO) and (**B**) 6-gingerol (6-GIN).

The roots or rhizomes of ginger (*Zingiber officinale* Roscoe; family: Zingeberaceae) have been used as dietary supplements since ancient times [1]. Although it is cultivated in several countries around the globe, India and China are the leading producers of ginger [1,2]. In recent years, ginger has gained attention from researchers around the globe due to its broad range of therapeutic activities, in addition to its low toxicity [1,2,3]. The main therapeutic activities of ginger are antioxidant [3], anti-inflammatory [4], anti-apoptotic [5], analgesic [4], cytotoxic [5], anti-proliferative [5,6], antitumor [6], and anti-platelets [7] activities. The therapeutic activities of ginger are due to the presence of biomarker compounds, such as various gingerols and shogaols [6,7]. The most abundant gingerols of ginger rhizome are 6-gingerol (6-GIN), 8-GIN, and 10-GIN [1,7]. Among shogaols, the most abundant is 6-shogaol (6-SHO) [6]. The chemical structures/formulae of 6-SHO and 6-GIN are presented in Figure 1.

A single ultra-violet (UV) spectrometry method was reported for the determination of 6-GIN in ginger extract [8]. Various high-performance liquid chromatography (HPLC) methods were reported for the determination of 6-GIN in plant extracts, commercial food products, and commercial formulations [1,2,9,10,11,12,13,14]. Different liquid chromatography mass-spectrometry (LC-MS) methods were applied for the analysis of 6-GIN, either alone or in combination with other ginger compounds in plant extracts and commercial formulations [15,16,17,18,19]. Some high-performance thin layer chromatography (HPTLC) methods have also been reported for the determination of 6-GIN in ginger extract, commercial foods, and commercial formulations [20,21,22]. The HPTLC method had also been utilized for the simultaneous determination of 6-GIN, 8-GIN, and 10-GIN in ultrasonication-assisted extract of ginger [23]. The HPTLC technique was also applied for the determination of a similar compound 8-GIN in plant extracts, commercial foods, and commercial formulations [24]. Some HPLC methods have been applied for the simultaneous determination of 6-SHO and 6-GIN in ginger extract and commercial formulations [25,26,27,28]. A HPLC method was also proposed for the simultaneous determination of 6-SHO and 6-GIN in human plasma samples [29]. A LC-MS method was applied for the simultaneous determination of 6-SHO and 6-GIN in ginger extract [30]. Although various analytical methodologies have been reported for the determination of 6-GIN or the simultaneous determination of 6-SHO and 6-GIN in ginger extracts, commercial foods, and commercial formulations, none of them used green analytical methodology. Moreover, reported HPTLC methods have also used toxic solvents in their mobile phase [20,21,22,23]. In addition, most of the reported HPTLC methods are based on normal phase chromatography [20,24]. Few green HPTLC methods, utilizing the same mobile phase (ethanol and water), have been reported for the determination of rivaroxaban, delafloxacin, and diosmin, but the method application and analyzed compounds are different, with respect to the present method [31,32,33]. Some HPTLC methods have also been reported for the determination of bioactive compounds, such as vanillin and flavonoids in plant extracts, but they used toxic mobile phase in addition to different analyzed compounds [34,35]. Recently, the analytical methods associated with green analytical chemistry or environmentally benign analytical chemistry have increased significantly in literature [31,36,37].

Based on reported literature, it was found that green HPTLC methods have not been reported for the analysis of 6-SHO or 6-GIN alone or in their combination. Moreover, green reversed phase HPTLC (RP-HPTLC) methods offer many advantages over conventional HPTLC methods, such as the avoidance of nonpolar fractions, avoidance of interference of the impurities, formation of compact spots, detection clarity, and non-toxicity to the environment [36,37,38]. Hence, the aim of this study was to develop and validate a green RP-HPTLC method for the simultaneous determination of 6-SHO and 6-GIN in traditional and ultrasonication-assisted extracts of ginger rhizome, commercial ginger powder, commercial capsules, and commercial ginger teas for the first time. The proposed green RP-HPTLC method for the simultaneous determination of 6-SHO and 6-GIN was validated for linearity, accuracy, precision, robustness, sensitivity, and specificity, as per International Conference on Harmonization (ICH) Q2 (R1) recommendations [39].

## 2. Materials and Methods

### 2.1. Materials

Standard 6-SHO, 6-GIN, and commercial ginger powder were obtained from Natural Remedies (Bangalore, India). HPLC grades of methanol and ethanol were acquired from E-Merck (Darmstadt, Germany). Ginger commercial capsules and ginger roots containing teas were obtained from the local market in Riyadh, Saudi Arabia. Ginger rhizomes were purchased from the local market in Al-Kharj, Saudi Arabia. HPLC grade water was collected from the Milli-Q unit. All the solvents were of chromatography grades and other reagents were of analytical reagent grades.

### 2.2. Instrumentation and Analytical Conditions

Simultaneous RP-HPTLC-densitometry analysis of 6-SHO and 6-GIN was carried out using the following instrumentation and analytical conditions:

HPTLC apparatus: CAMAG TLC system (CAMAG, Muttenz, Switzerland)

Software: WinCAT’s (version 1.4.3.6336, CAMAG, Muttenz, Switzerland)

Syringe for sample application: CAMAG microliter Syringe (Hamilton, Bonaduz, Switzerland)

TLC plate: 10 × 20 cm glass backed plates pre-coated with RP silica gel 60 F254S plates (E-Merck, Darmstadt, Germany)

Sample applicator: CAMAG Linomat-V (CAMAG, Muttenz, Switzerland)

Gas for sample application: nitrogen

Development chamber: CAMAG automatic developing chamber 2 (ADC2) (CAMAG, Muttenz, Switzerland)

TLC scanner: CAMAG TLC scanner-III (CAMAG, Muttenz, Switzerland)

Stationary phase: 10 × 20 cm glass backed plates pre-coated with RP silica gel 60 F254S plates (E-Merck, Darmstadt, Germany)

Mobile phase for 6-SHO and 6-GIN: ethanol:water (6.5:3.5 *v*/*v*)

Saturation time: 30 min at 22 °C

Development distance on plate: 80 mm

Development mode: linear ascending mode

Sample application rate: 150 nL/s

Densitometry of scanning mode: absorbance/reflectance.

Scanning wavelength for 6-SHO and 6-GIN: 200 nm

### 2.3. Calibration Curve of 6-SHO and 6-GIN

The stock solution (SS) of 6-SHO and 6-GIN was prepared separately by dissolving an accurately weighed 10 mg of 6-SHO and 6-GIN in 10 mL of methanol. About 1 mL of SS of 6-SHO and 6-GIN was diluted further using mobile phase to obtain the final SS of 100 μg/mL. Serial dilutions of SS of 6-SHO and 6-GIN were made by taking different volumes of 6-SHO SS or 6-GIN SS and diluting them with mobile phase to obtain concentrations in the range of 100–700 ng/band for 6-SHO and 50–600 ng/band for 6-GIN. The SSs of 6-SHO and 6-GIN were prepared in six replicates (*n* = 6). Around 200 μL of each concentration of 6-SHO and 6-GIN was applied on TLC plates, and the peak area was recorded. The calibration curve (CC) of 6-SHO and 6-GIN was obtained by plotting the concentrations against the measured areas of 6-SHO and 6-GIN. The CCs for 6-SHO and 6-GIN were obtained for six replicates (*n* = 6).

### 2.4. Extraction Procedure for Ginger Rhizomes

Accurately weighed 5 g of the dried whole rhizomes of ginger (*Zingiber officinale* Roscoe) were refluxed with methanol (100 mL) for 1 h in a water bath and filtered through Whatman filter paper (No. 41). The marc left out was refluxed again three times with 70 mL of methanol for 1 h and filtered. The solvent was evaporated using a rotary vacuum evaporator, and the residue was dissolved in 50 mL methanol in a volumetric flask. This procedure was performed in three replicates (*n* = 3). This solution was used as a test solution in the RP-HPTLC‒densitometry analysis of 6-SHO and 6-GIN in the methanolic extract of ginger rhizome.

### 2.5. Extraction Procedure from Commercial Ginger Powder, Capsules, and Ginger Teas

For the simultaneous determination of 6-SHO and 6-GIN in commercial dietary supplement capsules, five soft gelatin capsules containing ginger powder were opened, transferred in a beaker, and mixed to ensure that a homogenous sample was obtained. The ginger root teas and ginger powder were also transferred in a beaker separately. About 1 g of each of ginger root dietary supplement capsule, ginger powder, and tea were weighed and transferred to separate beakers. They were then extracted thrice with 70 mL of methanol separately. Filtrates were combined and concentrated using a rotary vacuum evaporator to a final volume of 10 mL. Each procedure was performed in three replicates (*n* = 3). The obtained solutions were used as test solutions in the RP-HPTLC analysis. The amount of 6-SHO and 6-GIN was determined in both of the test solutions, using the RP-HPTLC-densitometry method.

### 2.6. Ultrasonic Extraction Procedure for Whole Rhizomes of Ginger

The ultrasonic extraction of the dried whole rhizomes of ginger was carried out by ultrasonic vibrations (ultrasound-assisted extraction) using the Bransonic series (Model CPX5800H-E; New Jersey, NJ, USA). A total of 5 g of dried whole rhizomes of ginger was taken and extracted with 100 mL of methanol. The solvent was evaporated using a rotary vacuum evaporator and the residue was dissolved in 50 mL methanol in a volumetric flask. It was ultrasonicated at 50 °C for 1 h. This procedure was performed in three replicates (*n* = 3). This solution was used as a test solution for the simultaneous determination of 6-SHO and 6-GIN in an ultrasound-assisted extract of ginger rhizomes.

### 2.7. Ultrasonic Extraction Procedure from Commercial Ginger Powder, Capsules, and Ginger Teas

For the simultaneous determination of 6-SHO and 6-GIN in commercial dietary supplement capsules, five capsules containing ginger powder were opened, transferred in a beaker, and mixed to ensure that a homogenous sample was obtained. The ginger teas and ginger powder were also transferred in separate beakers. About 1 g each of ginger root dietary supplement capsule, ginger powder, and tea were weighed and transferred to separate beakers. It was then ultrasonicated thrice at 50 °C for 1 h with 70 mL of methanol separately. Filtrates were combined and concentrated using a rotary vacuum evaporator to a final volume of 10 mL. Each procedure was performed in three replicates (*n* = 3). The obtained solutions were used as test solutions in the RP-HPTLC analysis. The amount of 6-SHO and 6-GIN in ultrasonic-assisted extracts of commercial capsules and ginger root teas was determined using the RP-HPTLC-densitometry method.

### 2.8. Method Validation

The proposed green RP-HPTLC-densitometry method for the simultaneous determination of 6-SHO and 6-GIN was validated for linearity, precision, accuracy, robustness, sensitivity, and specificity, according to ICH Q2 (R1) recommendations [39]. The linearity of 6-SHO and 6-GIN was obtained by plotting the concentration of 6-SHO and 6-GIN against their respective HPTLC responses. The linearity for 6-SHO was studied in the range of 100–700 ng/band in six replicates (*n* = 6). However, the linearity for 6-GIN was recorded in the range of 50–600 ng/band in six replicates (*n* = 6). The method accuracy for 6-SHO and 6-GIN was determined as the percent of recovery (% recovery) using the standard addition method. The previously analyzed solutions of 6-SHO (100 ng/band) and 6-GIN (100 ng/band) were spiked with extra 0, 50, 100, and 150% amounts of 6-SHO and 6-GIN. The resultant concentrations of 100, 150, 200, and 250 ng/band for 6-SHO and 6-GIN were re-analyzed using a green RP-HPTLC-densitometry method in six replicates (*n* = 6). The % recovery was calculated at each concentration of 6-SHO and 6-GIN.

Method precision for 6-SHO and 6-GIN was determined as repeatability and intermediate precision. Repeatability (intra-day precision) for 6-SHO and 6-GIN was evaluated by the analysis of samples on the same day at 100, 150, 200, and 250 ng/band concentrations in six replicates (*n* = 6). However, intermediate (inter-day precision) for 6-SHO and 6-GIN was evaluated using the analysis of samples on three different days at 100, 150, 200, and 250 ng/band concentrations in six replicates (*n* = 6).

Method robustness for 6-SHO and 6-GIN was determined by introducing some minor modifications in the composition of green mobile phase during 6-SHO and 6-GIN analysis. The original mobile phase composition of ethanol:water (6.5:3.5) was changed into ethanol:water (6.7:3.3) and ethanol:water (6.3:3.7) for the positive and negative level, respectively. The robustness for 6-SHO and 6-GIN was evaluated in six replicates (*n* = 6).

Method sensitivity for 6-SHO and 6-GIN was determined as the limit of detection (LOD) and limit of quantification (LOQ) by adopting the standard deviation (SD) method. The LOD and LOQ values for 6-SHO and 6-GIN were calculated in six replicates (*n* = 6) using the following equations:

(1) $$\mathrm{LOD}=3.3\times \frac{\mathrm{SD}}{S}$$

(2) $$\mathrm{LOQ}=10\times \frac{\mathrm{SD}}{S}$$

where S is the slope of the CC of 6-SHO or 6-GIN.

Method specificity for 6-SHO and 6-GIN was evaluated by comparing the retardation factor (R_f_) values and UV-absorption spectra of 6-SHO and 6-GIN in the ginger rhizome extract, commercial capsules, and ginger root teas with that of standard 6-SHO and 6-GIN.

### 2.9. Application of a Green RP-HPTLC Method in the Simultaneous Analysis of 6-SHO and 6-GIN in Ginger Rhizome Extract, Commercial Ginger Powder, Capsules, and Ginger Teas

The obtained samples of traditional and ultrasonication-assisted extracts of ginger rhizome, commercial ginger powder, capsules, and ginger teas were applied on RP-HPTLC plates, and their chromatograms were obtained under the same experimental conditions and procedures as described for the simultaneous quantification of standard 6-SHO and 6-GIN. The HPTLC area of 6-SHO and 6-GIN in all studied test samples was recorded in three replicates (*n* = 3). The amount of 6-SHO and 6-GIN in all studied test samples was computed using the respective CC of 6-SHO and 6-GIN.

### 2.10. Statistical Analysis

All the values are expressed as the mean ± SD of three or six replicates. The statistical analysis was carried out by applying Dunnett’s test, using GraphPad Prism software (version 6, GraphPad, San Diego, CA, USA). This analysis was performed at 5% level of significance.

## 3. Results and Discussion

### 3.1. Method Development

Based on available reports in literature, it was found that no green RP-HPTLC method reported for the simultaneous determination of 6-SHO and 6-GIN in plant extracts, food products, and formulations. Hence, this study was carried out to develop and validate a green RP-HPTLC method for the simultaneous quantification of 6-SHO and 6-GIN. In this study, the green mobile phase was obtained by the simple mixture of ethanol and water (green solvents) in comparison to the normal HPTLC method. The application of RP-HPTLC methods offer several advantages over normal phase HPTLC methods [37,40]. In addition, green RP-HPTLC methods for the simultaneous determination of 6-SHO and 6-GIN also reduce the toxicity of the proposed method to the environment [35,36].

In the proposed research, different compositions of ethanol and water, such as 5:5 (%, *v*/*v*), 6:4 (%, *v*/*v*), 7:3 (%, *v*/*v*), and 6.5:3.5 (%, *v*/*v*), were investigated as the green mobile phases for the development of a suitable band for the simultaneous RP-HPTLC-densitometry analysis of 6-SHO and 6-GIN. All studied mobile phases were developed using chamber saturation conditions.

**Figure 2:**
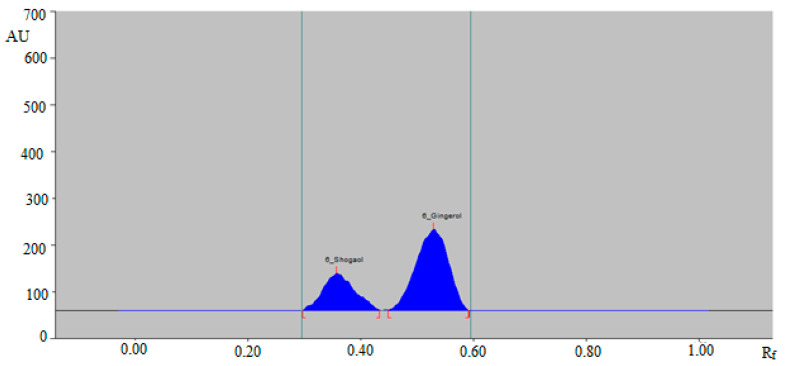
High-performance thin layer chromatography (HPTLC)-densitogram of standard 6-SHO and 6-GIN.

From the results, it was found that the mixtures of ethanol and water, such as 5:5 (%, *v*/*v*), 6:4 (%, *v*/*v*), and 7:3 (%, *v*/*v*), presented poor densitometry peaks of 6-SHO and 6-GIN with poor symmetry. When the mixture of ethanol and water, such as 6.5:3.5 (%, *v*/*v*), was investigated, it was found that this combination presented well-resolved, symmetrical, and compact densitometry peaks of 6-SHO at R_f_ = 0.36 ± 0.01 and of 6-GIN at R_f_ = 0.53 ± 0.01 (Figure 2).

Based on these results, the mixture of ethanol: water 6.5:3.5 (%, *v*/*v*) was optimized as the green mobile phase for the simultaneous analysis of 6-SHO and 6-GIN in traditional and ultrasonication-assisted extracts of ginger rhizome, commercial ginger powder, capsules, and ginger teas. The band spectra for 6-SHO and 6-GIN were obtained in the densitometry mode, and the maximum response under reflectance/absorbance mode was obtained at the wavelength (λ_max_) = 200 nm for 6-SHO and 6-GIN. Therefore, all simultaneous analyses of 6-SHO and 6-GIN were performed at λ_max_ = 200.

### 3.2. Method Validation

Different validation parameters for the simultaneous analysis of 6-SHO and 6-GIN were evaluated based on ICH guidelines [39]. The results for linear regression analysis of CCs of 6-SHO and 6-GIN are tabulated in Table 1.

The CC of 6-SHO and 6-GIN was found to be linear in the range of 100–700 and 50–600 ng/band, respectively. The results indicated a good linear relationship between the concentration and response of 6-SHO and 6-GIN. The value of determination coefficient (R^2^) for 6-SHO and 6-GIN was computed as 0.9988 and 0.9995, respectively. The obtained R^2^ values for 6-SHO and 6-GIN were significant (*p* < 0.05). Overall, the results of linear regression analysis indicated that a green RP-HPTLC method was linear for the simultaneous quantification of 6-SHO and 6-GIN.

Method accuracy for 6-SHO and 6-GIN was computed as % recovery, and results are summarized in Table 2.

The % recoveries of 6-SHO and 6-GIN after spiking an extra 0–150% were obtained as 98.8–101.6 and 99.0–101.5%, respectively, using a green RP-HPTLC method. The percent of relative standard deviation (% RSD) values in the recovery of 6-SHO and 6-GIN were obtained as 1.10–1.46 and 1.07–1.39%, respectively. The obtained % recoveries within the limit of 100 ± 2% for 6-SHO and 6-GIN indicated that the green RP-HPTLC-densitometry method was accurate for the simultaneous quantification of 6-SHO and 6-GIN. Method precision for 6-SHO and 6-GIN was computed as % RSD, and results are summarized in Table 3.

The % RSD values of 6-SHO and 6-GIN for the intraday precision were calculated as 0.8–1.5 and 0.7–1.0%, respectively. The % RSD values of 6-SHO and 6-GIN for inter-day precision were obtained as 0.7–1.6 and 0.7–1.0%, respectively. The obtained values of % RSD for 6-SHO and 6-GIN within the range of ±2% showed that the green RP-HPTLC method was precise for the simultaneous quantification of 6-SHO and 6-GIN.

Robustness for 6-SHO and 6-GIN was determined by introducing minor modification in the composition of mobile phase, and results are summarized in Table 4. The errors (% RSD) for 6-SHO and 6-GIN after introducing minor modification in the composition of mobile phase were obtained as 0.7–0.8 and 0.6–0.8%, respectively. The small variations in R_f_ values and lower % RSD values suggested that the green RP-HPTLC method was robust for the simultaneous determination of 6-SHO and 6-GIN.

The method sensitivity for 6-SHO and 6-GIN was evaluated in terms of LOD and LOQ, and results are summarized in Table 1. The LOD and LOQ values for 6-SHO were calculated as 33.65 ± 0.84 and 100.95 ± 2.52 ng/band, respectively. However, the LOD and LOQ values for 6-GIN were calculated as 16.84 ± 0.36 and 50.52 ± 1.08 ng/band, respectively. The obtained values of LOD and LOQ indicated that the green RP-HPTLC method was sensitive enough for the simultaneous determination of 6-SHO and 6-GIN.

**Figure 3:**
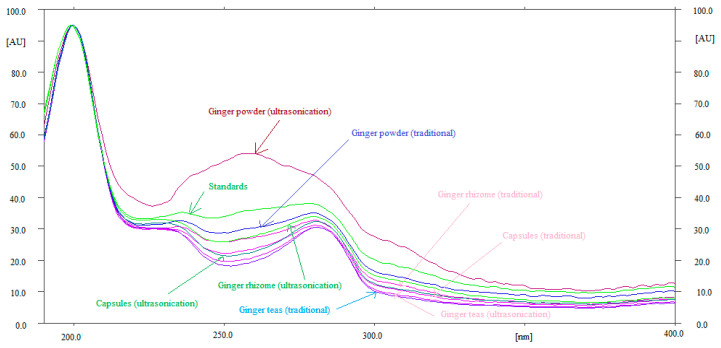
Overlaid ultra-violet (UV) absorption spectra of standards (6-SHO and 6-GIN), ginger rhizome, ginger powder, commercial capsules, and ginger teas extracted from traditional and ultrasonication methods.

Method specificity and the peak purity of 6-SHO and 6-GIN were evaluated by comparing the overlaid UV-absorption spectra of 6-SHO and 6-GIN in ginger rhizome extract, commercial powder, capsules, and ginger root teas with that of the standard. The overlaid UV spectra of standard 6-SHO and 6-GIN and 6-SHO and 6-GIN in traditional and ultrasonication-assisted extracts of ginger rhizome, commercial ginger powder, capsules, and ginger teas are shown in Figure 3. Although the UV spectra of 6-SHO and 6-GIN in ultrasonication-assisted extract of ginger powder was quite different, their maximum responses were recorded as λ_max_ = 200 nm. The quite different spectra of 6-SHO and 6-GIN in ultrasonication-assisted extract of ginger powder could be due to the presence of other compounds. The maximum densitometry response of 6-SHO and 6-GIN in standards and traditional and ultrasonication-assisted extracts of ginger rhizome, commercial ginger powder, capsules, and ginger root teas were found at λ_max_ = 200 nm under the reflectance/absorbance mode. The similar UV-absorption spectra, R_f_ values, and λ_max_ of 6-GIN and 6-SHO in standard and traditional and ultrasonication-assisted extracts of ginger rhizome, commercial ginger powder, capsules, and ginger root teas suggested the method specificity for the simultaneous determination of 6-SHO and 6-GIN.

### 3.3. Application of the Green RP-HPTLC Method in Simultaneous Analysis of 6-SHO and 6-GIN in Ginger Rhizome Eextract, Commercial Ginger Powder, Capsules, and Ginger Teas

**Figure 4:**
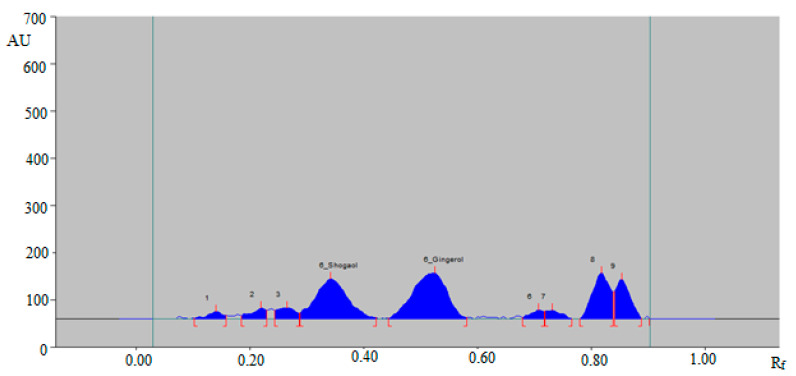
HPTLC-densitogram of 6-SHO and 6-GIN in traditional ginger rhizome extract.

A green RP-HPTLC method could be an alternative approach of conventional HPTLC techniques for the simultaneous determination of 6-SHO and 6-GIN in traditional and ultrasonication-assisted extracts of ginger rhizome, commercial ginger powder, capsules, and ginger teas. The densitometry peaks of 6-SHO and 6-GIN from traditional and ultrasonication-assisted extracts of ginger rhizome, commercial ginger powder, capsules, and ginger teas were verified by comparing their single TLC spot at R_f_ = 0.36 ± 0.01 for 6-SHO and R_f_ = 0.53 ± 0.01 for 6-GIN with that of standards 6-SHO and 6-GIN. The representative HPTLC densitogram of 6-SHO and 6-GIN in traditional ginger rhizome extracts is shown in Figure 4, which shows similar peaks of 6-SHO and 6-GIN to that of standard 6-SHO and 6-GIN. In addition, seven additional peaks were also recorded in the traditional ginger rhizome extract.

**Figure 5:**
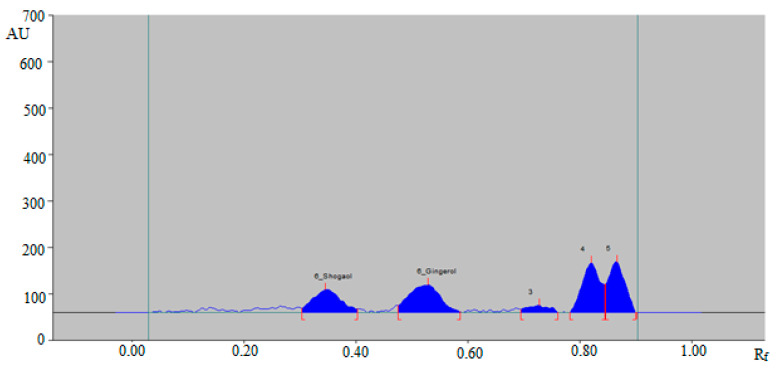
HPTLC-densitogram of 6-SHO and 6-GIN in traditional commercial ginger powder extract.

The representative HPTLC densitogram of 6-SHO and 6-GIN in traditional commercial ginger powder extract is shown in Figure 5, which also shows similar peaks of 6-SHO and 6-GIN to that of standard 6-SHO and 6-GIN. In addition, three additional peaks were recorded in traditional commercial ginger powder extract.

**Figure 6:**
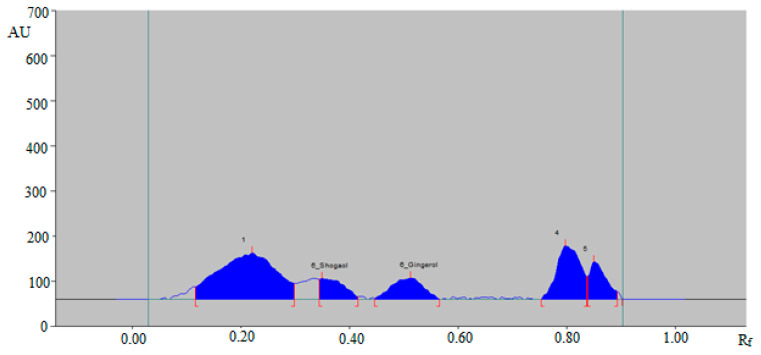
HPTLC-densitogram of 6-SHO and 6-GIN in traditional commercial capsules extract.

The representative HPTLC densitogram of 6-SHO and 6-GIN in traditional commercial capsule extract is shown in Figure 6, which also suggests similar peaks of 6-SHO and 6-GIN to that of standard 6-SHO and 6-GIN. In addition, three additional peaks were found in the traditional commercial capsule powder extract.

The presence of additional peaks in all studied traditional and ultrasonication-assisted extracts indicated that the green RP-HPTLC method could be successfully utilized for the simultaneous determination of 6-SHO and 6-GIN in the presence of other compounds or impurities. The amount of 6-SHO and 6-GIN in traditional and ultrasonication-assisted extracts of ginger rhizome, commercial ginger powder, capsules, and ginger teas was determined by the CCs of 6-SHO and 6-GIN. The amount of 6-SHO in traditional extracts of ginger rhizome, commercial ginger powder, capsules, and ginger teas was found as 12.1 ± 0.8, 17.9 ± 0.9, 10.5 ± 0.4, and 9.6 ± 0.3 mg/g of extract, respectively. However, the amount of 6-SHO in ultrasonication-assisted extracts of ginger rhizome, commercial ginger powder, capsules, and ginger teas was found as 14.6 ± 0.7, 19.7 ± 1.0, 11.6 ± 0.4, and 10.7 ± 0.4 mg/g of extract, respectively. The amount of 6-GIN in traditional extracts of ginger rhizome, commercial ginger powder, capsules, and ginger teas was recorded as 10.2 ± 0.6, 15.1 ± 0.8, 7.3 ± 0.2, and 6.2 ± 0.2 mg/g of extract, respectively. Meanwhile, the amount of 6-GIN in ultrasonication-assisted extracts of ginger rhizome, commercial ginger powder, capsules, and ginger teas was found as 12.7 ± 0.7, 17.8 ± 0.8, 8.8 ± 0.3, and 7.9 ± 0.3 mg/g of extract, respectively. In general, the amount of 6-SHO and 6-GIN was found to be significantly higher in traditional and ultrasonication-assisted extracts of commercial ginger powder and ginger rhizome compared with traditional and ultrasonication-assisted extracts of commercial capsules and ginger teas (*p* < 0.05). In addition, the amount of 6-SHO and 6-GIN in ultrasonication-assisted extracts was higher than traditional extracts. The amount of 6-SHO and 6-GIN in ultrasonication-assisted extracts of commercial ginger powder and ginger rhizome was significantly higher than their traditional extracts (*p* < 0.05). However, the amount of 6-SHO and 6-GIN in ultrasonication-assisted extracts of commercial capsules and ginger teas was not significant to their traditional extracts (*p* > 0.05). Based on these results, the ultrasonication method for the extraction of 6-SHO and 6-GIN in different ginger products was considered superior over the traditional method of extraction. Overall, these results suggested that the green RP-HPTLC method can be successfully applied in the simultaneous determination of 6-SHO and 6-GIN in a wide variety of food and pharmaceutical products containing 6-SHO and 6-GIN as the active constituents.

### 3.4. Literature Comparison

The green RP-HPTLC method for the simultaneous determination of 6-SHO and 6-GIN was compared with different analytical methods reported for the simultaneous determination of 6-SHO and 6-GIN. The results of comparison are tabulated in Table 5.

Different validation parameters, such as linearity range, accuracy, and precision of the green RP-HPTLC method, were compared with literature methods. The linearity range, accuracy, and precision of the reported LC-MS method for the simultaneous determination of 6-SHO and 6-GIN were found to be much inferior to the green RP-HPTLC method [29]. In addition, the linearity range, accuracy, and precision of various reported HPLC methods for the simultaneous determination of 6-SHO and 6-GIN were also found to be inferior to the green RP-HPTLC method [24,25,27]. HPTLC methods have not been reported for the simultaneous determination of 6-SHO and 6-GIN or 6-SHO alone. However, various HPTLC methods have been reported for the determination of 6-GIN alone or in combination with other compounds [20,21,22,23]. Therefore, the green RP-HPTLC method was also compared with reported HPTLC methods for the determination of 6-GIN alone. The results of this comparative analysis are tabulated in Table 6. The accuracy and precision for the determination of 6-GIN of most of the reported HPTLC methods were found within the limit of ICH guidelines [36]. However, the accuracy of the HPTLC method reported by Khan et al. was outside the limit of the ICH guidelines [23]. The accuracy of the present HPLTLC method was superior to those reported by Khan et al. [23]. However, there were not many differences found in the accuracy or precision of the present green RP-HPTLC method with other reported methods [20,21,22,24]. 

On the other hand, the linearity range (50–1000 ng/band) of one reported HPTLC method was wider than the present RP-HPTLC method (50–600 ng/band) with similar lower limit detections [20]. The linearity range (140–840 ng/band) of the other reported HPTLC method was slightly wider than the present RP-HPTLC method (50–600 ng/band), but its lower limit detections were much higher than the present HPTLC method [23]. The linearity range (50–500 ng/band) of the other reported HPTLC method was slightly narrower than the present RP-HPTLC method (50–600 ng/band), with similar lower limit detections [24]. The lower limit of detections of the other two HPTLC methods were much lower than the present RP-HPTLC method [21,22]. Overall, both reported methods, as well as the present HPTLC method, were found to be suitable for the determination of 6-GIN. However, the reported HPTLC methods were not utilized for the simultaneous determination of 6-SHO and 6-GIN. Overall, the present RP-HPTLC method for the simultaneous determination of 6-SHO and 6-GIN was found to be more simple, accurate, precise, cost-effective, and sensitive than reported analytical methods.

## 4. Conclusions

Due to the lack of green RP-HPTLC methods for the simultaneous determination of 6-SHO and 6-GIN in literature, the objective of this study was to develop and validate a green RP-HPTLC method for the simultaneous determination of 6-SHO and 6-GIN in traditional and ultrasonication-assisted extracts of ginger rhizome, commercial ginger powder, capsules, and ginger teas for the first time. The green RP-HPTLC is simple, accurate, precise, robust, sensitive, and specific for the simultaneous determination of 6-SHO and 6-GIN. The amount of 6-SHO and 6-GIN was found to be higher in ultrasonication-assisted extracts of ginger rhizome, commercial ginger powder, capsules, and ginger teas than their respective traditional extracts. Based on these results, ultrasonication has been proposed as the preferred method for the extraction of 6-SHO and 6-GIN from ginger rhizome, commercial ginger powder, capsules, and ginger teas over the traditional method of extraction. Overall, the results of this study suggested that the green RP-HPTLC method can be successfully utilized in the simultaneous analysis of 6-SHO and 6-GIN in the real samples of ginger food and pharmaceutical products having 6-SHO and 6-GIN as the active constituents.

## Figures and Tables

**Table 1:** Linear regression analysis data for the calibration curve (CC) of 6-shogaol (6-SHO) and 6-gingerol (6-GIN) for the green reversed phase high-performance thin layer chromatography (RP-HPTLC) method (mean ± SD; *n* = 6).

Parameters	6-SHO	6-GIN
Linearity range (ng/band)	100–700	50–600
Regression equation	Y = 9.04x + 53.78	Y = 13.22x + 693.37
R^2^	0.9988	0.9995
Slope ± SD	9.04 ± 0.54	13.22 ± 1.08
Intercept ± SD	53.78 ± 2.86	693.37 ± 15.75
Standard error of slope	0.22	0.44
Standard error of intercept	1.16	6.42
95% confidence interval of slope	8.09–9.99	11.33–15.12
95% confidence interval of intercept	48.76–58.81	665.70–721.03
LOD ± SD (ng/band)	33.65 ± 0.84	16.84 ± 0.36
LOQ ± SD (ng/band)	100.95 ± 2.52	50.52 ± 1.08

LOD: limit of detection and LOQ: limit of quantification.

**Table 2:** Accuracy data of 6-SHO and 6-GIN for the green RP-HPTLC method (mean ± SD; *n* = 6).

Excess Drug Added to Analyte (%)	Theoretical Content (ng)	Conc. Found (ng) ± SD	Recovery (%)	RSD (%)
6-SHO				
0	100	98.8 ± 1.4	98.8	1.4
50	150	148.5 ± 2.0	99	1.3
100	200	202.4 ± 2.6	101.2	1.2
150	250	254.1 ± 2.8	101.6	1.1
6-GIN				
0	100	101.5 ± 1.4	101.5	1.3
50	150	152.1 ± 1.9	101.4	1.2
100	200	198.3 ± 2.3	99.1	1.1
150	250	247.6 ± 2.6	99	1

RSD: relative standard deviation.

**Table 3:** Precision data of 6-SHO and 6-GIN for the green RP-HPTLC method (mean ± SD; *n* = 6).

Conc. (ng/band)	Repeatability (Intraday Precision)			Intermediate Precision (Inter-Day)		
	Area ± SD	Standard Error	RSD (%)	Area ± SD	Standard Error	RSD (%)
6-SHO						
100	956 ± 15	6.1	1.5	963 ± 16	6.5	1.6
150	1402 ± 17	7.1	1.2	1428 ± 17	7.2	1.2
200	1912 ± 18	7.5	0.9	1888 ± 16	6.5	0.8
250	2396 ± 20	8.4	0.8	2363 ± 18	7.4	0.7
6-GIN						
100	2098 ± 21	8.7	1	2115 ± 21	8.9	1
150	2618 ± 24	9.8	0.9	2686 ± 25	10.2	0.9
200	3412 ± 27	11.3	0.8	3378 ± 25	10.5	0.7
250	4156 ± 32	13.2	0.7	4082 ± 29	12.1	0.7

**Table 4:** Robustness data of 6-SHO and 6-GIN for the green RP-HPTLC method (mean ± SD; *n* = 6).

Conc. (ng/band)	Mobile Phase Composition (Ethanol:Water)			Results		
	Original	Used		Area ± SD	% RSD	R_f_
6-SHO						
		6.7:3.3	0.2	1503 ± 13	0.8	0.35
150	6.5:3.5	6.5:3.5	0	1432 ± 11	0.8	0.36
		6.3:3.7	−0.2	1342 ± 9	0.7	0.37
6-GIN						
Mobile phase composition (ethanol:water)						
		6.7:3.3	0.2	2753 ± 22	0.8	0.52
150	6.5:3.5	6.5:3.5	0	2654 ± 19	0.7	0.53
		6.3:3.7	−0.2	2513 ± 15	0.6	0.54

**Table 5:** Comparison of the current green RP-HPTLC method with reported analytical methods for the simultaneous determination of 6-SHO and 6-GIN.

Analytical Method	Compound						Ref.
	6-SHO			6-GIN			
	Linearity Range	Accuracy (% Recovery)	Precision (% RSD)	Linearity Range	Accuracy (% Recovery)	Precision (% RSD)	
LC-MS	1–40 (µg/mL)	83–110	2.0–4.0	5.5–220 (µg/mL)	87–100	2.0–8.0	[29]
HPLC	1–5 (µg/mL)	97.8–100.8	0.4–1.5	1.0–5.4 (µg/mL)	97.8–100.8	0.4–1.5	[24]
HPLC	6–18 (µg/mL)	84.7–92.8	3.0	20–60 (µg/mL)	91.5–102.3	3.4	[25]
HPLC	10–250 (µg/mL)	99.8–101.1	0.2–1.6	10–250 (µg/mL)	99.3–99.7	0.4–1.5	[27]
HPTLC	100–700 (ng/band)	98.8–101.6	0.7–1.6	50–600 (ng/band)	99.0–101.5	0.7–1.0	Present work

**Table 6:** Comparison of the current green RP-HPTLC method with reported HPTLC methods for the determination of 6-GIN.

Analytical Method	Linearity Range (ng/Band)	Accuracy (% Recovery)	Precision (% RSD)	Ref.
HPTLC	50–1000	98.1–98.8	0.6–1.2	[20]
HPTLC	140–840	95.6–101.4	0.7–1.4	[23]
HPTLC	150–900	98.2–99.6	0.0–0.1	[21]
HPTLC	100–1400	99.7–100.1	1.0–1.4	[22]
HPTLC	50–500	98.2–99.2	0.4–1.6	[24]
HPTLC	50–600	99.0–101.5	0.7–1.0	Present work
